# Gravure Printing of Graphite-Based Anodes for Lithium-Ion Printed Batteries

**DOI:** 10.3390/membranes12100999

**Published:** 2022-10-14

**Authors:** Maria Montanino, Anna De Girolamo Del Mauro, Claudia Paoletti, Giuliano Sico

**Affiliations:** 1 ENEA Italian National Agency for New Technologies, Energy and Sustainable Economic Development, Portici Research Centre, 80055 Portici, Italy; 2 ENEA Italian National Agency for New Technologies, Energy and Sustainable Economic Development, Casaccia Research Centre, 00123 Roma, Italy

**Keywords:** gravure printing, printed batteries, anodes, lithium-ion batteries, multilayer, graphite

## Abstract

Aimed at the growing interest in printed batteries, widely used industrial gravure printing was recently proven to be able to produce high-quality electrodes for lithium-ion batteries (LiBs), demonstrating its utility in the study of new functional materials. Here, for the first time, gravure printing was investigated for the mass production of well-known low-cost graphite-based anodes for LiBs. Graphite was also chosen as a case study to explore the influence of process parameters on the layer microstructure and the performance of the printed anodes. In particular, upon decreasing the size of the active material nanoparticles through ball-milling, an enhancement in anode performance was observed, which is related to an improvement in the material distribution in the printed layer, even in the case of increasing mass loading through a multilayer approach. A further improvement in performance, close to the theoretical capacity, was possible by changing the ink parameters, obtaining a denser microstructure of the printed anode. Such good results further demonstrate the possibility of using gravure printing for the mass production of electrodes for printed batteries and, in general, components in the field of energy.

## 1. Introduction

Printing technologies can fulfill several tasks related to the most recent demands on industrial battery manufacturing. Contrary to deposition methods that are not solution-based and/or roll-to-roll compatible, the main benefits of printing are the lower cost and large-scale production, while compared to conventional solution-based coating techniques, printing can also offer many advantages such as high speed, fine control of the layer characteristics, high resolution and the possibility to produce any desired shape and pattern [1,2,3,4]. Moreover, in the frame of the European Union (EU) proposal for a new regulation to make battery technology and industrial production more sustainable [5], roll-to-roll compatible printing techniques can represent a suitable solution to enhance the sustainability requirement of the battery production process.

Recently, printing has been increasingly investigated, especially in the field of a new class of storage devices, printed batteries, which can be highly customizable to perfectly fit the devices to be powered. Printed batteries are devices with a volume below 10 mm^3^ and specific capacities of 5–10 mAh/cm^3^ [6]. Such devices are of growing interest, since they are more and more involved in our daily lives, e.g., in portable and wearable electronics, biomedical application and the internet of things (IoT) [7]. To date, the few industrially produced printed batteries are mainly fabricated via screen printing and are limited to non-rechargeable devices [6].

Among the printing techniques, widely used industrial gravure printing appears very appealing for the cost-effective production of functional films, especially in the field of energy, thanks to its ability to combine high speed (up to 400 m/min) and high printing quality (resolution below 0.2 µm) [8,9,10]. The gravure is a roll-to-roll large-area printing technique able to produce films of every shape with a very limited waste of energy and materials [11]. In particular, the material wastage limitation is attributed to the large-scale production process (in-line) while most of the used deposition technologies are small-area and/or batch processes resulting in waste. Despite its many advantages, to date, such a technique is seldom investigated in the field of printed batteries, mainly due to the difficulties in obtaining a proper mass loading using the necessary diluted inks, which are fundamental in the case of electrode production. In addition, printing composite materials while keeping the functionality of the layer remains a challenge.

Overcoming such limits, thanks to the use of a multilayer approach able to increase the active material’s areal mass loading [12], we recently demonstrated the possibility of employing gravure printing in the manufacturing of electrodes for lithium-ion batteries (LiBs), producing layers of desired mass loading with very good performance in the devices [4,13]. Moreover, gravure printing was found to be useful in investigating the arrangement of the materials in layer formation and its consequences on layer performance, allowing to exploit most of the properties of the employed materials. Nevertheless, when a new material is used, a new ink formulation has to be investigated and adequate process parameters have to be determined.

In this work, for the first time, we used gravure printing for producing high-quality graphite-based anodes for LiBs, investigating the microstructure-performance relationships of the anodic printed layer. Graphite was chosen as a case study due to its wide use, low cost and high chemical stability [7,14,15]. However, it can be challenging for an active material to show its theoretical capacity due to the limitations of the manufacturing process [14]. In fact, in most cases in which a material is used in the form of film, layer manufacturing plays a fundamental role in its functionality and efficiency.

The proposed anodes involved a water-based binder, and water was used as the main solvent in the ink formulation, in response to the most recent green requests on highly sustainable and safe manufacturing and improving the energy efficiency [5,11].

## 2. Materials and Methods

The inks were prepared using the following materials at fixed weight percentages as dry content: graphite (from Linyi Gelon, Shandong, China) as the active material (88%), sodium carboxy methyl cellulose (CMC, from Panreac Quimica, Castellar del Vallès, Spain) as the binder (6%), super P (by Thermo Fisher, Waltham, MA, USA) as the conductive carbon (6%). Demineralized water was used as prevalent solvent. 2-propanol (IPA, by Merck KGaA, Darmstadt, Germany) was employed as a process co-solvent, and mixed with water in different proportions to improve the printability. The inks were printed on corona-pre-treated Cu foils, with 10 μm of thickness, using an IGT G1-5 printer equipped with a cylinder with a line density of 40 lines/cm, stylus angle of 120°, cell depth of 72 µm and screen angle of 53°. Several preliminary printing tests, including changing the ink concentration, the solvent ratio, the ink preparation and several printing parameters, made it possible to individuate the best process parameters, as reported below. In some cases, the dry components were mixed with water and ball-milled for three hours to reduce and homogenize the particulate size of the starting materials. More layers of the same ink were overlapped to obtain a final layer with a mass loading suitable for printed batteries. After the printing of each layer, fast drying was performed using nitrogen blowing. The overall layers were finally dried at 100 °C for one hour. No calendaring was performed on the printed anodes. Scanning electron microscopy (1530, LEO Elektronenmikroskopie GmbH, Oberkochen, Germany) was used to investigate the morphology of the printed anodes. The gravure-printed anodes were cut into disks 1 cm in diameter and tested as electrodes in a half cell versus lithium metal through charge and discharge cycles, at constant and variable rates, using Maccor (Tulsa, OK, USA) equipment; we used as an electrolyte 1M LiPF_6_ in a 3:7 (wt:wt) mixture of ethylene carbonate (EC) and diethyl carbonate (DEC).

## 3. Results and Discussion

**Figure 1:**
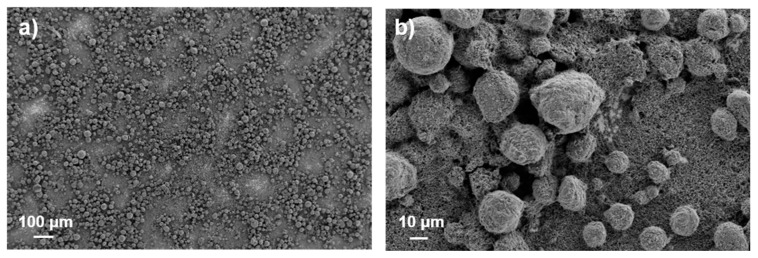
SEM images of the 5L anode microstructure obtained by printing ink at 25 wt% of dry content (**a**) ×150 and (**b**) ×1400.

Gravure printing has a complex fluid-dynamic involving different stages depending on several physical–chemical parameters of the ink and of the substrate. Such parameters, together with the process parameters, determine the final quality of the printed layer [16]. Despite its complex physical nature, gravure printing can provide a simple approach for the scalable manufacturing of high-quality functional layers [17]. The inks suitable for gravure printing have low viscosity (1–100 mPa s) involving a large amount of solvent [16]. To improve the sustainability of the process, a water-soluble binder and water were used as prevalent solvents in the ink formulation. On the other hand, to improve the ink printability, 2-propanol was used as a co-solvent, decreasing the high surface tension of the water-based solution, as also reported elsewhere [4]. In particular, for achieving good printability, the ink must have a surface tension below the surface energy of the substrate and of the printing cylinder. To this end, corona treatment was used to increase the copper surface energy to about 50 mN/m, while the addition of IPA decreased the high surface tension of the water-based ink from 72 mN/m to about 30 mN/m, improving the wettability of both the cylinder (42 mN/m) and the substrate. The process parameters (speed and printing force) were experimentally adjusted while evaluating the printing quality in terms of macroscopic defect absence. Based on such evaluations, the proper combination of the process parameters was found to print anodic layers on copper substrate. The desired mass loading was obtained by overlapping layers of the same ink at fixed concentrations to simplify the overall process. Inks containing different dry contents, ranging from 15 to 30 wt%, were used for printing tests, using a mixed solvent of water and 2-propanol (80–20 wt%). Among them, the ink containing 25 wt% of dry content showed the best printability at a printing force of 700 N and a printing speed of 36 m/min, and was used to realize a five-layer (5L) anode. However, the microstructure of the final anode was not homogenous, showing large particles of graphite and small particles of carbon, resulting in a rough distribution of the materials and a poor substrate coverage (see Figure 1).

**Figure 2:**
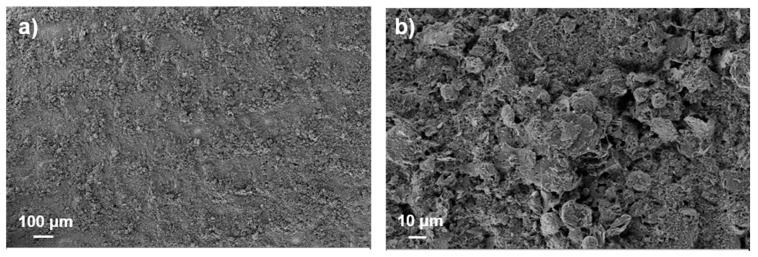
SEM images of the 5L anode microstructure obtained by printing ball-milled ink for three hours at 25 wt% of dry content (**a**) ×150 and (**b**) ×1400.

**Figure 3:**
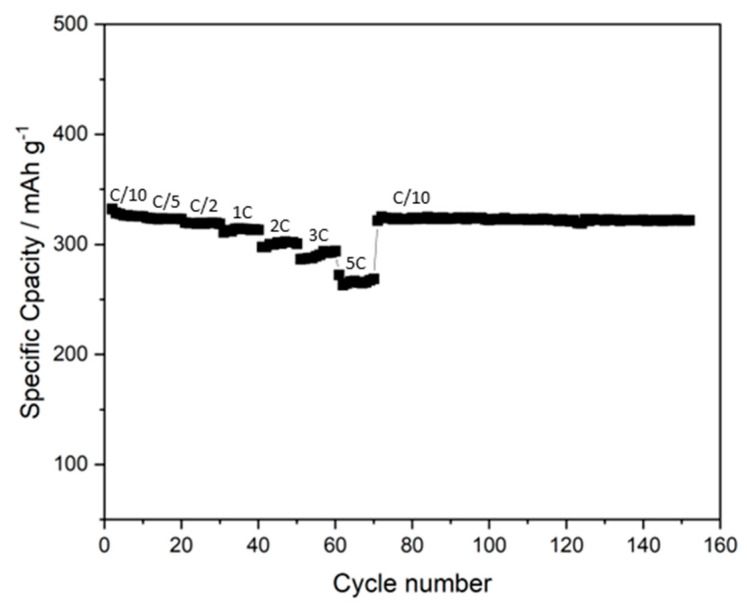
Discharge-specific capacity of the 5L anode obtained by printing ball-milled ink for three hours at 25 wt% of dry content at variable and constant rates.

To improve the distribution of the materials in the overall anodic layer, a 3 h ball-milling process was used in the ink preparation. A 5L anode was prepared using this method, printing under the same conditions as previously mentioned; the obtained anode showed improved substrate coverage and improved distribution of the materials due to the decrease in the initial size of the graphite (see Figure 2). The particle morphology changed from spherical to platelet-like and the printed layer showed good performance (see Figure 3). However, a decrease in the ink stability was observed, probably due to the IPA’s capability to agglomerate in the case of disaggregated particles. In fact, the ink viscosity was shown to increase during the multilayer printing, reaching a viscosity not suitable for gravure.

**Figure 4:**
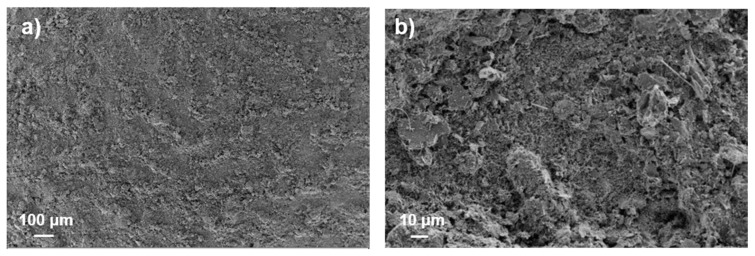
SEM images of the 6L anode microstructure obtained by printing ball-milled ink for three hours at 18 wt% of dry content using 90–10 % water–IPA solvent (**a**) ×150 and (**b**) ×1400.

To further improve the substrate coverage and the ink stability, decreasing in the ink concentration and IPA content was tried in order to improve the printability. An ink containing the 18 wt% of dry content was prepared, keeping constant the percentage of the components and the ball-milling conditions, while the IPA content was decreased and a mix of water–IPA of 90–10 wt% was used for the ink preparation. Such ink does not show agglomeration phenomena and was used to realize a six-layer anode (6L): the number of layers was increased from 5 to 6 to balance the decrease in the ink concentration and to keep the mass loading approximatively constant (see Table 1). The SEM image shows improved coverage, keeping a similar microstructure (see Figure 4).

**Figure 5:**
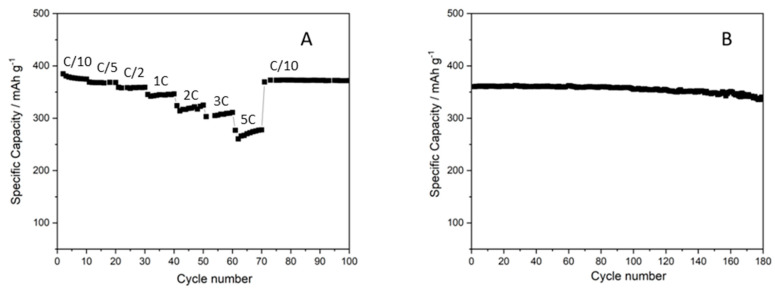
Discharge-specific capacity of the 6L anode microstructure obtained by printing ball-milled ink for three hours at 18 wt% of dry content using 90–10% water–IPA solvent at (**A**) variable and (**B**) C/10 constant rates.

Different morphology of the graphite generally implies differences in its activity as anodic material [15], but the performance of the 6L anode (see Figure 5) increased with respect to that of the ball-milled 5L anode, even if the particles had the same morphology (see Figure 2 and Figure 4), suggesting better particle distribution and, consequently, improved interconnection of the layer components. Thus, both the IPA concentration and the change in the dry ink content played a fundamental role in the ink deposition and, consequently, on the morphology of the layer and its performance. The decrease in the IPA content from 20 to 10 wt% did not strongly affect the surface tension but drastically reduced the agglomeration phenomenon of the carbon-based materials. As a consequence, the printability was improved by the use of IPA, while the ink stability was enhanced by the decrease in IPA content, affecting the homogeneity of the ink in the printed layer. On the other hand, the dry content affected the lay-down of the materials on the substrate, influencing their arrangement in the overall layer, especially in case of multilayering. Decreasing the dry content improved the lay-down and the layer distribution due to the increase in the solvent amount (from 75 to 82 wt%), also improving the superimposition of materials in the overlapping of subsequent layers. This process allowed us to obtain similar results compared to a layer commonly densified via calendaring, skipping the post-processing step and resulting in simplification of the overall process. Moreover, the gravure printing process provided highly homogeneous particle distribution, enhancing the stability and the life cyclability of the anode, preventing dendrite formation and premature electrode degradation [14]. Ideally, the electrode should be fully dense, even keeping a certain flexibility to properly allow for mechanical expansion and contraction due to the lithiation–delithiation [17]. The improvement in the ink preparation and the optimization of the ink printing parameters led to very good particle arrangement of the printed layer being reached, obtaining a capacity close to the theoretical one (372 mAh/g) [18]. As a consequence, improvements in the capacity, efficiency, stability and long life cyclability were observed (see Figure 5). When the charging rate is increased up to 2C (namely, charging in 30 min), the maximum observed decrease in capacity is below 14%, with a specific capacity exceeding 300 mAh/cm^2^ (see Figure 5); after cycling at increasing rates, the performance of the optimized anode recovers the starting capacity value obtained at C/10, showing good stability even when cycled at different rates. Gravure printing was able to produce very homogeneous layers, as well as surface characteristics, producing a very fine controlled and spikeless interface with the electrolyte, thus generating a very uniform SEI (Solid Electrolyte Interface). The lack of preferential accumulation of active material at the interface prevented the consequent local accumulation of materials during the charge–discharge process (lithiation–delithiation), thus avoiding dendrite formations. Proof of such behavior was the observed long cycle life, keeping constant performance (without capacity fading) for up to 180 cycles. Such results confirm the possibility of involving industrial gravure printing in the cost-effective production of printed batteries, skipping the calendaring and obtaining performance close to the theoretical one, with high stability and a long cycle life.

## 4. Conclusions

The possibility of using highly sustainable gravure printing for the large-scale and cost-effective production of anodes for LiBs was verified, and gravure-printed graphite-based anodes were successfully produced for the first time. The multilayer approach allowed us to obtain the required mass loading. The correlation between the layer micro-structure and its performance was investigated. The introduction of ball-milling and the optimization of the ink preparation allowed us to improve the layer quality in terms of homogeneity and material distribution. Thus, an improvement in the anodic performance was observed, approaching the theoretical capacity and showing a long cycle life. Due to the good performance, the calendaring step was skipped with simplification of the overall process. This study proved the feasibility of gravure printing for anode production and highlighted the importance of the process parameters in the layer characteristics and performance. These results, combined with the previous results on gravure-printed cathodes, promote highly scalable gravure printing as a viable industrial method for the printed battery manufacture, even from the perspective of fully printed devices.

## Figures and Tables

**Table 1:** Characteristics of the gravure-printed anodes.

Layer n.	Ink Dry Content (wt%)	Ball-Milling	Overall Active Material (mg cm^−2^)	Final Layer Thickness (µm)	Final Layer Density (g cm^−3^)
5	25	no	0.90	27	0.33
5	25	yes	1.10	21	0.52
6	18	yes	0.85	18	0.47

## Data Availability

The raw data can be obtained from the corresponding author upon reasonable request.
